# Chemical shift transfer: an effective strategy for protein NMR assignment with ARTINA

**DOI:** 10.3389/fmolb.2023.1244029

**Published:** 2023-10-03

**Authors:** Henry Wetton, Piotr Klukowski, Roland Riek, Peter Güntert

**Affiliations:** ^1^ Institute of Molecular Physical Science, ETH Zurich, Zurich, Switzerland; ^2^ Institute of Biophysical Chemistry, Goethe University Frankfurt, Frankfurt, Germany; ^3^ Department of Chemistry, Tokyo Metropolitan University, Hachioji, Japan

**Keywords:** NMR, machine learning, automated spectra analysis, automated assignment, ARTINA, FLYA, protein

## Abstract

Chemical shift transfer (CST) is a well-established technique in NMR spectroscopy that utilizes the chemical shift assignment of one protein (source) to identify chemical shifts of another (target). Given similarity between source and target systems (e.g., using homologs), CST allows the chemical shifts of the target system to be assigned using a limited amount of experimental data. In this study, we propose a deep-learning based workflow, ARTINA-CST, that automates this procedure, allowing CST to be carried out within minutes or hours of computational time and strictly without any human supervision. We characterize the efficacy of our method using three distinct synthetic and experimental datasets, demonstrating its effectiveness and robustness even when substantial differences exist between the source and target proteins. With its potential applications spanning a wide range of NMR projects, including drug discovery and protein interaction studies, ARTINA-CST is anticipated to be a valuable method that facilitates research in the field.

## 1 Introduction

Recently, ARTINA (Klukowski et al., 2022), the first workflow that automates the analysis of protein NMR data for signal identification, resonance assignment (Schmidt and Güntert, 2012), and structure determination (Güntert et al., 1997; Güntert and Buchner, 2015) was developed, demonstrating the capability of machine learning to advance biomolecular NMR. With ARTINA, the entire NMR data analysis process can be completed on a web server (Klukowski et al., 2023) without human supervision and within hours after the NMR measurements are completed, replacing weeks or months of human labor. The procedure requires spectra with an information content that is sufficient to unambiguously assign chemical shifts without any prior knowledge about the protein structure. The exact amount of experimental data required for *de novo* chemical shift assignment with ARTINA depends on the signals’ resolution and signal-to-noise ratio. Typically, the requirement for the measurement time on expensive NMR spectrometers scales to approximately one or two weeks.

Often, one would like to study a system by NMR that is similar to an already assigned protein (Thompson et al., 2012). Examples of this include homologous proteins (Redfield and Robertson, 1991; Bartels et al., 1996), proteins studied under different experimental conditions (e.g., temperature, pH value, ligand concentration) (Jang et al., 2012; Banelli et al., 2017; Zieba et al., 2018), or proteins with bound ligands and in apo form (Orts and Gossert, 2018; Laveglia et al., 2021; Plata et al., 2023). In such cases, it is desirable to use knowledge about the known system, in particular its assigned chemical shifts, as complementary input for the automated analysis of a related target system with ARTINA. Such complementary input typically makes it possible to assign chemical shifts or determine the protein structure using a smaller set of spectra. In this work we evaluate the accuracy of ARTINA-CST and develop guidelines to utilize our method for chemical shift transfer applications.

## 2 Methods

### 2.1 Chemical shift transfer

The interface required for chemical shift transfer (CST) is included within the FLYA algorithm used by ARTINA for chemical shift assignment (Schmidt and Güntert, 2012). The function of this algorithm has been described in detail by Schmidt and Güntert; in brief, a list of expected peaks is constructed from the protein of interest’s amino acid sequence, then this list is mapped to a list of measured peaks generated by manual or automated peak picking. The mapping is improved iteratively using global and local optimization methods (Bartels et al., 1997). This procedure is repeated for a series of replicates and the final output is determined as a majority consensus of these.

**FIGURE 1:**
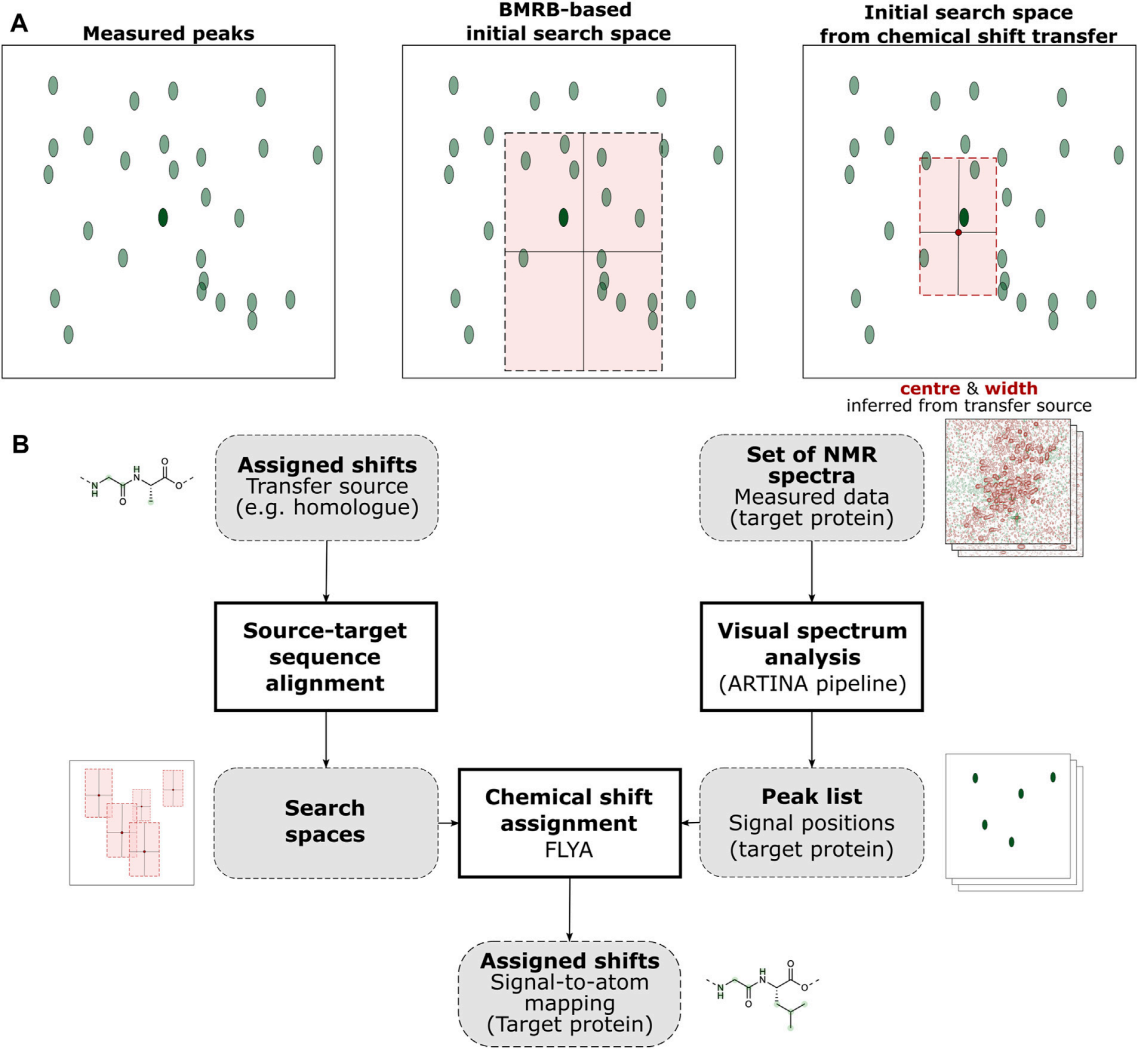
Graphical abstract of the ARTINA-CST workflow. **(A)** CST affects automated chemical shift assignment through the selection of peak search spaces (light red rectangle). Without CST, the search space is estimated based on statistical information extracted from the BMRB database, whereas with CST, it is determined from a previously assigned source protein (the dark red circle indicates the chemical shifts of the source protein, and the highlighted area is specified by a user-defined parameter). The resulting area is centered closer to the target peak of interest (dark green oval) and contains less incorrect peaks (light green ovals). **(B)** The chemical shifts of the source protein are prepared by sequence alignment to the target protein and subsequent extraction of search spaces from the chemical shifts of the aligned residues. Measured NMR data is then input to FLYA as a list of peaks extracted automatically from spectra, yielding the assigned shifts of the target protein.

In order to initialize the assignment of expected peaks to measured peaks, the default procedure within FLYA uses statistics from the Biological Magnetic Resonance Data Bank (BMRB) (Hoch et al., 2023) to construct an initial “search space” for each expected peak, from which a measured peak is picked at random to assign it (Schmidt and Güntert, 2012). For each atom in a protein, this search space is defined by a statistical distribution parametrized by the mean and standard deviation of chemical shifts of the given atom type over all occurrences of its amino acid type within the BMRB. Due to the scale of the database and the high number of peaks in protein NMR spectra, this usually generates a large area containing also many incorrect possibilities for assignment, the center of which can deviate from the peak’s true position. By improving the initial search space for each atom, i.e., reducing its size and shifting its center closer to the true position, the accuracy of FLYA assignments can therefore be improved significantly (Bartels et al., 1996; Aeschbacher et al., 2013). The essence of the CST procedure relies on this concept, aiming to provide a better estimate of the peak’s true position and therefore also allowing for a smaller search space size (Figure 1A).

CST is performed as follows (Figure 1B): Given a source protein with a known assignment of chemical shifts and a target protein whose shifts have yet to be assigned, first a mapping of source atoms to target atoms must be defined. In the trivial case where source and target protein are identical in sequence, each source atom can simply be mapped to its copy in the target. However, this need not always be the case, and transfer of assignment data between different protein samples could also be highly beneficial in many cases. In this work, we compared pairs of distinct proteins by first performing a local pairwise sequence alignment tuned to produce large aligned sub-sequences between a small number of wide gaps (Supplementary Table S1). Each identical pair of amino acids in this alignment could then be used for CST. Aligned pairs of different amino acids and amino acids aligned to gaps were simply disregarded, falling back to FLYA’s default setting.

Based on this source-target mapping, new initial search spaces can be defined for each atom in the target protein, centered around the corresponding chemical shift in the source. Since this new center is likely to be more accurate, i.e., closer to the true position of the atom’s shift than the mean value obtained from BMRB, the search space’s width can then be reduced (higher precision), further facilitating its assignment.

The determined search space centers and sizes are then entered into FLYA as a list of mean values and standard deviations (“chemical shift statistics”), with the algorithm automatically applying the default BMRB-based search space for any atom not included.

When using FLYA as part of the ARTINA pipeline, automated visual analysis of each spectrum generates the experimental peaks to be used as input (Klukowski et al., 2022). For this work, execution was stopped after obtaining an assignment from FLYA. In practice, however, the output assignments can also be passed to the next stages of ARTINA, e.g., structure determination.

### 2.2 NMR data

The dataset established for the training and testing of ARTINA models (Klukowski et al., 2022) was the source of raw NMR spectral data in this study. Since CST is most efficient if only a small number of spectra have to be measured for the target protein, we used a small subset of NMR spectra types from the ARTINA dataset, consisting only of the 2D HSQC ([^1^H, ^13^C]-HSQC, [^1^H, ^15^N]-HSQC) and 3D NOESY (^13^C-resolved [^1^H, ^1^H] NOESY, ^15^N-resolved [^1^H, ^1^H] NOESY) spectra. Unless specified otherwise, we used this small subset of spectra for a representative set of 15 proteins for all experiments in this work (Supplementary Table S2).

### 2.3 Test examples for automated chemical shift transfer

#### 2.3.1 Random perturbation

To evaluate the capabilities of ARTINA-CST, we initially generated CST test examples from the NMR data specified in section 2.2. Each test example was composed of the chemical shift list of the target (typically BMRB deposition) and source (randomly perturbed BMRB deposition) proteins, complemented by the set of NMR spectra of the target. The procedure ensured that each target protein had a corresponding source from which to draw chemical shift information, while also enabling quantification of the difference between the source and target shifts. The source chemical shift lists were created by manually perturbing deposited chemical shifts with Gaussian additive noise.

The standard deviation $\sigma_{i}$ of the normal distribution from which the perturbations were drawn was chosen individually for each atom i , using the standard deviation of the chemical shift over all occurrences of the same atom and amino acid type in the BMRB ( $\sigma_{i}^{\text{BMRB}}$ ):

$$\sigma_{i}=c\sigma_{i}^{\text{BMRB}}$$

where c is a scaling constant ( $c\in \left\{0.2,0.5,1.0\right\}$ ) selected to validate different experimental settings.

When applying this data for CST, the uncertainty value used to determine the search space width was set to the larger of either:

• the standard deviation used for perturbation ( $\sigma_{i}$ ), or
• a small tolerance of 0.04 ppm for ^1^H nuclei, and 0.4 ppm for both ^13^C and ^15^N.

To generate test examples, perturbations were applied to a specific fraction $p\in \left\{0.2,0.5,1.0\right\}$ of the total number of chemical shifts within each protein, selected at random.

By evaluating all combinations of the parameters p and c specified above, we conducted a total of nine distinct experiments. Each entailed a full assignment (backbone and sidechain) of the 15 benchmark proteins, resulting in a total of 135 assignments reported in the experimental section.

#### 2.3.2 Structure-based perturbation

The subsequent series of experiments incorporated protein structure information into the source shift list generation procedure. The routine was designed to replicate an experimental setting where chemical shifts change between source and target due to such factors as point mutations or ligand binding at specific sites. We employed a random approach like the one outlined in the previous section. However, the standard deviation of the perturbation was defined as a function of the distance from the “perturbation centers” ( G )—amino acids selected from the sequence of the target protein at random. The source chemical shift list generation procedure was the following:

1. For each perturbation center $g_{j}\in G$ and each atom $a_{i}$ in the protein structure S , calculate the distance ( $d_{ij}$ ) between the backbone amide nitrogen atoms of the perturbation center $g_{j}$ and the amino acid containing atom $a_{i}$ .
2. Calculate the decay factor $e_{i}$ for each atom $a_{i}$ , which combines the impact of all perturbation centers:

$$e_{i}=\sum_{j=1}^{\left|G\right|}f\left(d_{ij}\right)$$

where

$$f\left(x\right)=\left\{\begin{array}{c} 1\text{ if }x\leq x_{0}\\ 0\text{ if }x>x_{0} \end{array}\right.$$

is the step function parametrized by a distance cutoff $x_{0}$ .

3. Normalize each decay factor: $e_{i}^{\prime }=e_{i}\;/\max_{k\in S}e_{k}$ to ensure that $e_{i}^{\prime }$ is a scalar value in the range $\left[0,1\right]$ .
4. Calculate the standard deviation ( $\sigma_{i}$ ) of the additive noise for the i -th atom: $\sigma_{i}=e_{i}^{\prime }c\sigma_{i}^{\text{BMRB}}$ , where $c\in \left\{0.2,0.5,1.0\right\}$ is a constant used to validate different experimental settings.
5. Set the uncertainty value used to determine the search space width for the atom $a_{i}$ to the larger of either:
• the standard deviation used for perturbation ( $\sigma_{i}$ ), or
• a small tolerance of 0.03 ppm for ^1^H nuclei, and 0.4 ppm for both ^13^C and ^15^N.

**FIGURE 2:**
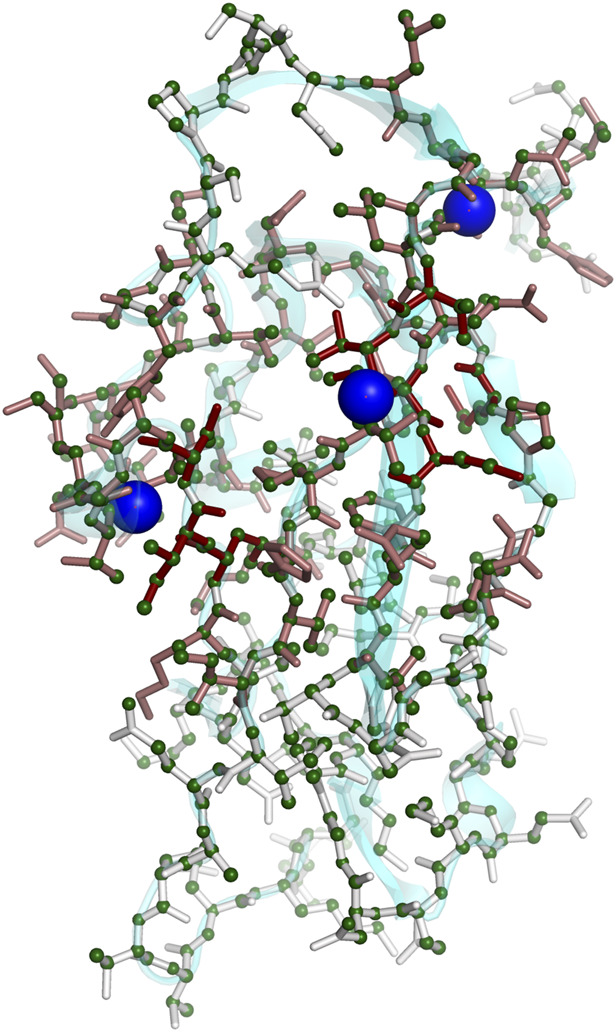
Three-dimensional rendering of decay factors ( $e_{i}^{\prime }$ ) using the protein PDB 1SE9 as an example. Three perturbation centers are represented by blue spheres that are overlaid with a light ribbon representation of the protein backbone in cyan. Decay factor values are presented for each residue as stick colors on a scale from 0 (white) to 1 (dark red). The resulting assignment accuracy is shown for each heavy atom as spheres, with assignment errors up to 0.4 ppm in dark green color. Incorrectly assigned heavy atoms are not present in this assignment result. For atoms with no visible sphere, automated assignments were made, however no reference manual shift was available for error calculation.

These steps form an alternative method to define the atom-specific chemical shift noise distribution $N\left(0,\sigma_{i}^{2}\right)$ , contrasting the fully randomized setting described in the preceding section. Aside from this distinction, both experimental settings follow the same logic. An example rendering of $e_{i}^{\prime }$ for three perturbation centers is shown in Figure 2.

In the experiment, we used the 15 benchmark proteins to generate 900 randomized CST test examples by drawing $x_{0}$ from a uniform distribution over the interval 5–25 Å, the number of perturbation centers from a categorical distribution over the integers 1–10, and positions of perturbation centers by selecting the specified number of protein residues from the sequence at random.

### 2.4 Homologous proteins

To evaluate CST with experimental data, we investigated sequence homology between proteins in the ARTINA dataset (Klukowski et al., 2022) (Supplementary Table S2) and both RefDB (Zhang et al., 2003) and BMRB using sequence alignment parameters specified in Supplementary Table S1. As sequence alignment scores had no upper bound, they were normalized by the score of aligning each protein to itself. If the homolog protein was found in both RefDB and BMRB, preference was given to RefDB. We refer to the protein from the ARTINA dataset as the target and from RefDB/BMRB as the source protein.

All pairs with normalized sequence alignment score above 80% were selected for chemical shift alignment. In this step we propagated information from the sequence alignment into the source chemical shift list. This was indispensable as differences between the source and target sequences, such as insertion or deletion, require appropriate reindexing of chemical shifts in the source before applying CST to the target.

Subsequently, each shift in the aligned source list was compared with the corresponding target shift. An aligned shift was considered “correct” if it was within a given tolerance from the target position (0.03 ppm for ^1^H, 0.4 ppm for ^13^C/^15^N). Then, we calculated the “fraction of correct aligned shifts” for each pair of source/target homologous proteins, which is defined as the ratio of correct aligned shifts to total shifts in the aligned list.

In this experiment, we used protein pairs with a fraction of correct aligned shifts greater than or equal to 50%. Combining with the requirement of >80% sequence alignment score, we identified 12 source-target pairs for which experimental spectra were available in the ARTINA benchmark. These pairs corresponded to 9 distinct target proteins (Supplementary Figure S2, Supplementary Table S3).

## 3 Results

### 3.1 Reference experiments

To investigate the impact of complementary input (chemical shift list of the source protein) on the accuracy of the chemical shift assignment of the target protein, we first evaluated the baseline performance of *de novo* automated assignments of target proteins, without any transfer information (Supplementary Figure S1).

Subsequently, we ran the chemical shift transfer in an idealized setting, where each target protein’s own chemical shift list was passed as the transfer source. This corresponds to the scenario, unlikely in practice, in which the chemical shifts do not change between the source and target proteins, or that the input shift list for CST is equal to the expected output. It is important to note that FLYA still uses peak lists prepared for a minimal set of experimental spectra ([^1^H, ^13^C]-HSQC, [^1^H, ^15^N]-HSQC, ^13^C-resolved [^1^H, ^1^H] NOESY, ^15^N-resolved [^1^H, ^1^H] NOESY) to perform chemical shift transfer. Therefore, it cannot simply copy the input shifts to the output, and some chemical shifts cannot be assigned due to missing signals in the input data. However, this experimental setting represents the best possible initial search space positions for FLYA, and therefore can be used to estimate an upper bound for CST accuracy. In this case, the width of each search space was set to 0.04 ppm for ^1^H nuclei and 0.4 ppm for ^13^C and ^15^N nuclei.

As expected, we observed a significant improvement in chemical shift accuracy by 23.5 percentage points (pp) in the idealized reference case, as compared to *de novo* assignment, where no complementary input was used. The average assignment accuracy of 68.8% for 15 proteins used in the *de novo* experiment increased to 92.3% using CST (Supplementary Figure S1). The fact that FLYA was unable to attain 100% accuracy in these reference experiments was likely mainly due to the minimal set of spectra used (Supplementary Table S2).

In both reference experiments (Supplementary Figure S1) the assignment errors had a tendency to accumulate in side-chains (90.6% accuracy with CST, 66.3% without), leaving the accuracy of the backbone assignment above the average (95.3% accuracy with CST, 73.4% without)—a result consistent with previous studies of FLYA (Schmidt and Güntert, 2012) and ARTINA (Klukowski et al., 2022).

Another notable observation is that the availability of source chemical shifts affects the variance of the output accuracy. In *de novo* experiments, the discrepancy between the most and least accurately assigned proteins is 34.9 pp, compared to only 13.8 pp in the idealized CST case.

Overall, the experiments we have conducted, ranging from scenarios with minimal to maximal information available for chemical shift transfer, indicate that source protein information provides useful guidance for the combinatorial optimization that reduces the ambiguity of the assignment, resulting in fewer errors and enhancing the overall reliability of protein assignment.

### 3.2 The impact of random and structure-based perturbations

To characterize ARTINA-CST under more realistic conditions, we carried out over 1,000 automated chemical shift transfers with 15 proteins using the test examples described in sections 2.3.1 and 2.3.2.

In the first series of experiments, a variable fraction of atoms was selected at random for perturbation (20%, 50% and 100%), as described in section 2.3.1, imitating a variable degree of discrepancies between known source chemical shifts and the target protein. Perturbed chemical shifts were used as input for the ARTINA-CST procedure together with 4 NMR spectra of the target protein ([^1^H, ^13^C]-HSQC, [^1^H, ^15^N]-HSQC, ^13^C-resolved [^1^H, ^1^H] NOESY, ^15^N-resolved [^1^H, ^1^H] NOESY). ARTINA-CST automatically extracted cross-peak positions from experimental data and combined them with the corresponding perturbed shift list, yielding the assignment of the target protein. This result was compared with *de novo* ARTINA assignment, which involved the same procedure, but without the perturbed chemical shift list as input.

**FIGURE 3:**
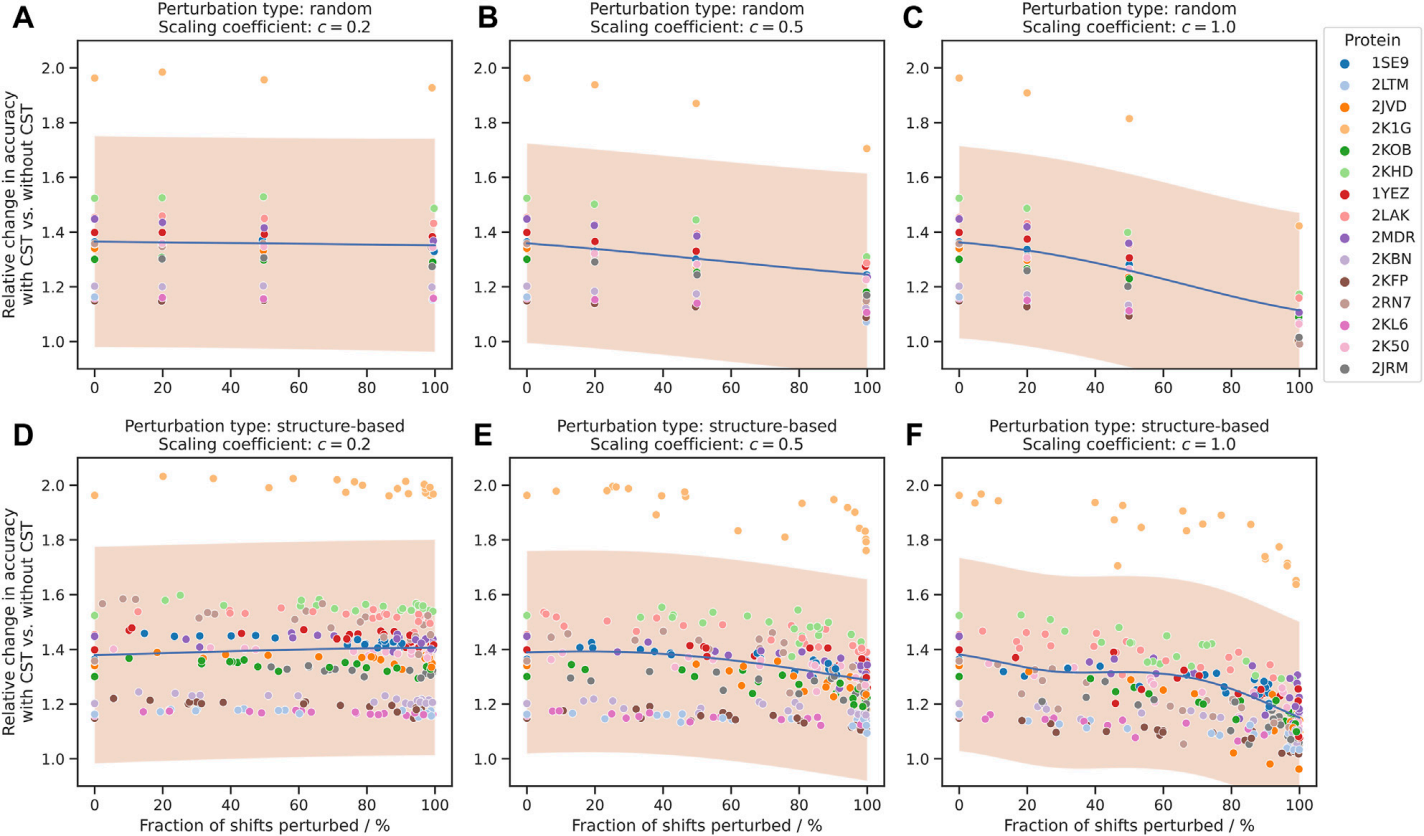
Improvement of chemical shift assignment with CST using randomly and structure-based perturbed shifts. On the vertical axis, the change in accuracy is given as the ratio between the chemical shift assignment accuracy with CST and corresponding *de novo* assignments without information about the source protein. On the horizontal axis, the fraction of shifts perturbed refers to all shifts where perturbations were applied, regardless of the size of the perturbation. The trend of each plot is modelled using Gaussian process regression and shown in blue, with a 95% confidence interval shown in orange. “Scaling coefficient” refers to the constant c used to scale the standard deviation of the applied perturbations (see sections 2.3.1 and 2.3.2). **(A–C)** Presents the results of the experiments with random and **(D–F)** with structure-based perturbations.

The use of CST turned out to be highly beneficial in all three experimental settings (20%, 50% and 100% shift perturbation) and for almost all proteins included in the study. The relative improvement in assignment accuracy is depicted in Figures 3A–C by the ratio between the accuracy of the chemical shift assignment with the CST procedure and with the *de novo* approach, with a value of 1.0 corresponding to a neutral effect of CST.

As expected, the value of the ratio depends on the parameters c and p of the test generation procedure (section 2.3.1). In the least challenging setting (Figure 3A), the scaling factor ( c=0.2 ) largely restricts chemical shift deviations between source and target chemical shift list. As the chemical shift values in the source and target shift lists lie in close proximity, even for perturbed atoms, the CST transfer procedure yields similar performance regardless of the number of atoms perturbed, demonstrating a 1.36-fold relative improvement on average as compared to the *de novo* runs.

When the scaling coefficient was set to a moderately higher value ( c=0.5 ), the effect of the larger of chemical shift perturbations on the overall CST accuracy was apparent (Figure 3B), with the relative improvement averaged over all proteins varying between 1.37 and 1.29 for 0% and 100% perturbed chemical shifts respectively. Finally, the strongest perturbation ( c=1.0 , Figure 3C) result in a further decrease in CST accuracy, as compared with previous settings (Figures 3A, B), but preserving strong positive impact (1.37-fold–1.23-fold improvement) relative to *de novo* calculations.

Subsequently, we repeated the above experiment using structure-based perturbations (section 2.3.2) instead of random ones. The results for these assignments show a strong resemblance to those for the fully randomized perturbations (Figures 3D–F), including correlation between CST accuracy and c , p parameters of the test example preparation procedure. The points with 0% perturbations correspond to the idealized case, as described in section 3.1.

Overall, we identified no major changes in the performance of the algorithm that depend on the spatial distribution of the chemical shift perturbation, and in both experimental settings the number of perturbations and corresponding variance were the primary factors affecting the accuracy of the procedure.

### 3.3 Case study with the lipoprotein Spr NlpC/P60 domain

For individual cases, we observed a particularly large improvement in the assignment obtained with the CST procedure. For instance, the C-terminal NlpC/P60 domain of lipoprotein Spr from *Escherichia coli* [PDB 2K1G (Aramini et al., 2008)], showed poor performance in *de novo* chemical shift assignment (48.3% accuracy), compared to 94.7% obtained in the idealized case of CST (Figure 3).

Even in the experimental setting with the strongest perturbation (standard deviation equal to BMRB standard deviation and perturbations applied to all shifts, which resulted in the large majority of shifts being moved significantly from their original positions), the fraction of correct assignments for this protein was raised by 20.4 pp upon application of the CST procedure. In all other cases the positive impact of the chemical shift transfer was even stronger (30.8–49.8 pp improvement, depending on the experimental setting).

The primary reason for such improvements was the ability of the CST method to resolve chemical shifts in the proximity of the dynamic loop of residues 16–32, 79–81, 90–93, 99–101, long positively charged side chains (37.3% without CST vs. 91.5% with idealized CST) and aromatics (54.9% vs. 93.7%).

### 3.4 Chemical shift transfer with homologous proteins

As described in section 2.4, we used pairs of homologous proteins identified in RefDB/BMRB and the ARTINA benchmark of NMR spectra to assess the performance of the chemical shift transfer in fully experimental setting. In this procedure, the chemical shift list deposited in RefDB/BMRB was regarded as source, and four NMR spectra ([^1^H, ^13^C]-HSQC, [^1^H, ^15^N]-HSQC, ^13^C-resolved [^1^H, ^1^H] NOESY, ^15^N-resolved [^1^H, ^1^H] NOESY) were used by ARTINA-CST as input for the transfer procedure to the target system. In this experiment all cross-peaks in the abovementioned spectra were identified automatically by deep neural network models included in the ARTINA visual spectrum analysis layer. As in the previous experiments, we performed for each target protein additional *de novo* assignments to assess the relative performance of the CST procedure.

**FIGURE 4:**
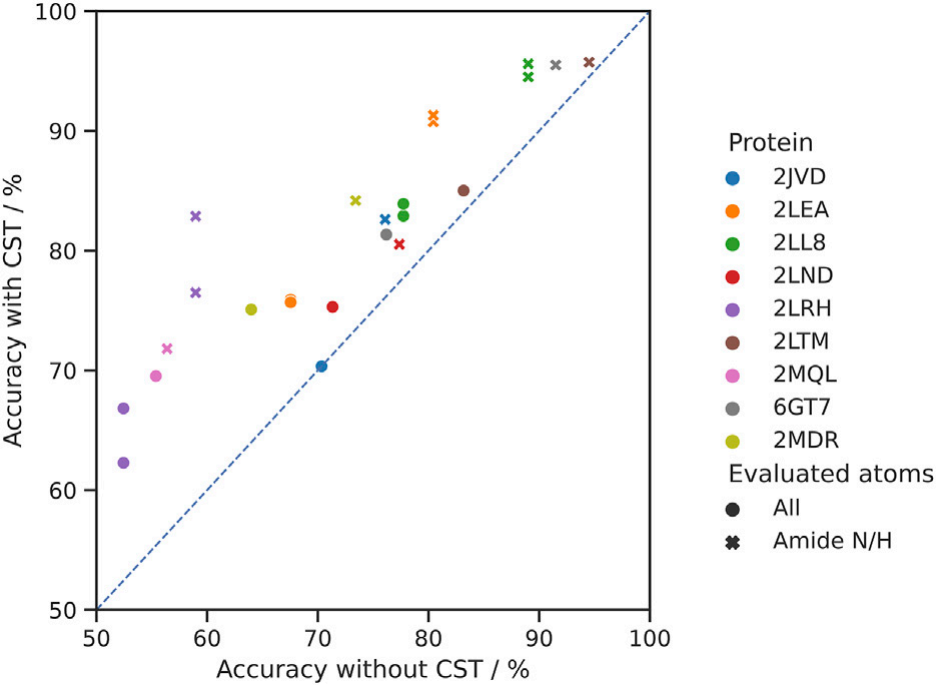
Comparison of the accuracy of the chemical shift assignment with and without CST. In the experiment 12 pairs of 9 homologous proteins were used. Each target protein is represented in a distinct color (for 2LEA, 2LL8 and 2LRH, two source proteins from BMRB were identified). Circles represent assignment accuracy over all atom types, whereas crosses signify the accuracy in the same experiments evaluated for the backbone amide N/H atoms only.

The results show an overall mean improvement by CST over *de novo* assignment of 7.4% (0.0%–14.4%) in the chemical shift assignment accuracy of all shifts and 9.7% (1.2%–23.9%) for the backbone NH groups (Figure 4). Out of 12 homolog pairs, the impact of CST was positive both for all shifts and NH groups in 11 cases. Only in one case (2JVD) the impact of CST was neutral for all shifts and positive for NH groups.

ARTINA-CST has a single parameter, $c_{\text{CST}}$ , which corresponds to the level of shift perturbation one expects to observe when transferring chemical shift assignments from the source to the target protein. It plays the same role as the scaling factor c for the synthetic data preparation in section 2.3.1, indicating the fraction of BMRB variance in individual shifts we expect to observe in a particular dataset. In this experiment, $c_{\text{CST}}$ was set to 1.0, indicating lack of a prior assumption about the variance of the distribution (i.e., we expect the chemical shifts in the target protein to deviate from the source the same way the individual shifts deposited in the BMRB database deviate from their mean value). Despite this conservative assumption, ARTINA-CST still yielded a substantial improvement in the chemical shift assignment accuracy, as compared with *de novo* assignment. Specific tuning of the expected perturbation parameter is expected to result in further improvement of the ARTINA-CST performance, as it provides weak constraints on the initial search-space for individual chemical shifts.

**FIGURE 5:**
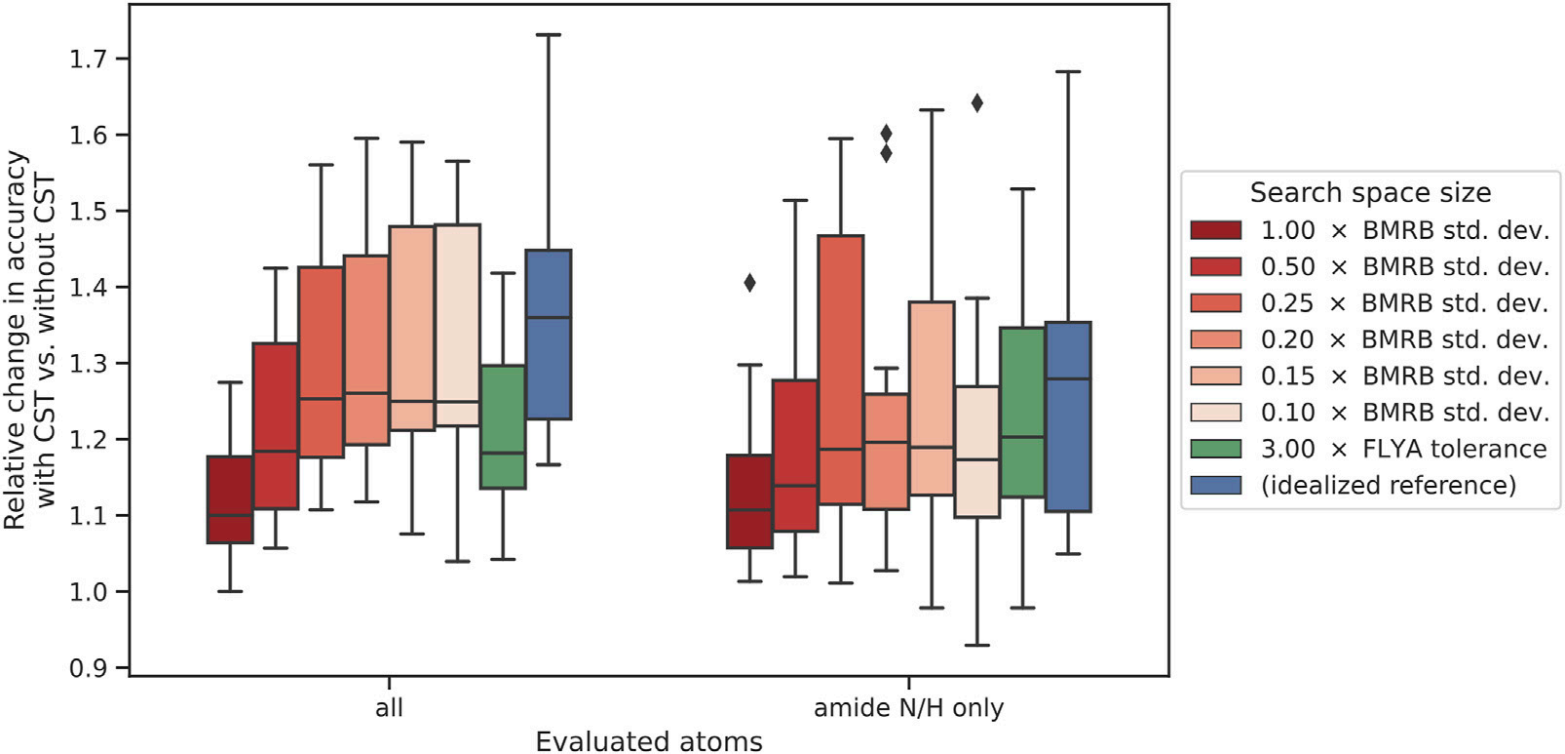
Impact of search space size on CST accuracy. The change in accuracy is given as the ratio between the accuracies with and without chemical shift transfer. The left-hand side represents assignment accuracy over all atom types, whereas the right-hand side signifies the accuracy in the same experiments only evaluated over all amide N/H atoms. Within each set of experiments, the furthest-right case (blue) represents the “ideal” reference experiment, using the target protein shift list as source for chemical shift transfer. The neighboring case (green) used a fixed-size search space of 0.09 ppm for ^1^H and 1.2 ppm for ^13^C/^15^N. The other boxes (red) represent a variable initial search space size calculated as a fraction of the BMRB standard deviation of chemical shifts.

In a second series of experiments, we therefore carried out chemical shift transfers between the 12 homolog pairs using different values of the expected perturbations, $c_{\text{CST}}$ = 0.10, 0.15, 0.20, 0.25, 0.50, 1.00, as well as with fixed-size search spaces of 0.09 ppm for ^1^H and 1.2 ppm for ^13^C/^15^N shifts that are independent of BMRB shift distributions. The results indicate that decreasing the size of the initial search space proved effective to increase ARTINA-CST accuracy (Figure 5). For each automated chemical shift assignment in this experiment, we calculated the ratio between the accuracies of CST-based and *de novo* assignment. With the lack of prior assumptions about the variance of chemical shifts ( $c_{\text{CST}}=1.0$ ), ARTINA-CST achieved 10% median improvement over *de novo* assignment, for which no information from the homologous protein was used. As the value of the expected perturbation parameter $c_{\text{CST}}$ decreased, the overall performance of the method increased and saturated at about 26% for $c_{\text{CST}}\in \left[0.1,0.25\right]$ . As two reference experiments, we evaluated the accuracy of chemical shift transfer with fixed tolerances (green box, Figure 5) and with an idealistic optimal reference, where the information about target chemical shifts is assumed to be known (upper bound accuracy) (blue box, Figure 5).

Chemical shift transfer impacts full assignment and amide N/H assignment in similar way (Figure 5). For $c_{\text{CST}}$ = 1.0, the relative median increase in assignment accuracy is 11%. The quality of the solution increases with decreasing expected perturbation coefficient, saturating at 20% for $c_{\text{CST}}\in \left[0.1,0.25\right]$ . The relative improvement for amide groups is smaller compared to all shifts, because the generally higher accuracy of NH shift assignments in *de novo* experiments leaves smaller room for improvements (68.0% and 77.2% accuracy for all shifts and NH groups respectively).

## 4 Discussion

An assigned set of chemical shifts establishes a basis for various studies in protein NMR spectroscopy. It facilitates structure elucidation, with chemical shifts being instrumental in determining hydrogen spatial contacts and providing insight into the three-dimensional architecture of the macromolecule. Beyond elucidating static structures, chemical shift assignments offer a unique window into protein dynamics, allowing for the monitoring of temporal changes, facilitating the observation of protein folding processes, conformational alterations, and molecular interactions. In the field of protein-ligand studies, the chemical shifts typically exhibit changes upon ligand binding, thereby pinpointing the site of interaction, as well as details regarding its molecular mechanism and affinity.

In this study we focused on chemical shift transfer–a technique that allows to find chemical shift assignment of a target protein of interest, given a (small) set of experimental spectra and the shift assignment of a similar source protein (e.g., a homolog). The technique is particularly suitable for studies of protein interactions, such as protein-ligand complexes, where information about the structure in apo form can be utilized to model the structure upon binding. Other applications include studies of protein mutations, where a wild-type structure with its assignment can be used as a source of the transfer for a series of mutants. Finally, chemical shift transfer finds its applications in studies of proteins under different physical conditions. In all experimental settings evaluated in this study, the goal of the chemical shift transfer was to find chemical shifts of the target protein with a small amount of experimental data, thereby reducing the measurement time from about 1 to 2 weeks to 2–3 days ([^1^H,^13^C]-HSQC, [^1^H,^15^N]-HSQC, and combined ^15^N,^13^C-resolved [^1^H,^1^H]-NOESY).

In this work we built upon our previous work with ARTINA (Klukowski et al., 2022) and FLYA (Schmidt and Güntert, 2012) to establish a fully automated workflow that performs chemical shift transfer automatically, strictly without any human involvement. Subsequently, we carried out over 1,000 automated chemical shift assignments to demonstrate the performance of ARTINA-CST approach and characterize its properties under different experimental settings.

We demonstrated the boundary performance of ARTINA-CST by carrying out automated CST with complete information about the target system and without any information from the homolog structure. Subsequently, we characterized the performance of our method, depending on such factors as the similarity of source and target protein chemical shifts, the variance of chemical shift perturbations, or the spatial distribution of chemical shift deviations. Finally, we demonstrated the performance of our approach using pairs of homolog proteins extracted from RefDB/BMRB databases, which have experimental data available in the ARTINA dataset. In all these experiments, we demonstrated the benefits of CST for the automated assignment of protein NMR spectra whenever appropriate data is available. Even in presence of large differences between the target protein and the source chemical shifts, CST allows an effective transfer of information to improve the assignment while requiring only a small set of spectra for the target protein. This experiment was carried out with a minimal set of NMR spectra for source-target pairs with at least 80% sequence homology (and correspondingly lower sequence identity). We expect CST to be possible also at even lower sequence similarity, where, however, it might be necessary to compensate for the larger number and size of the chemical shift differences by measuring one or more additional spectra (for instance, HNCO, HNCA, HNcoCA, or CBCAcoNH) for the target protein. On the other hand, we see the main practical applications of automated CST rather for source-target pairs with highly similar sequences such as orthologous proteins from different species and mutants in combination with temperature, pH, salt or other environment changes, ligand binding, *etc.*

Future improvements of ARTINA-CST are possible, provided that more NMR data relevant for chemical shift transfer is collected and deposited in public repositories. It would allow for the use of statistical methods or machine learning to characterize chemical shift perturbation patterns resulting from different types of transfers (e.g., changes of the physical conditions or ligand binding).

We believe that the method can find future applications in fundamental studies of proteins with NMR spectroscopy, including investigations of protein structure, dynamics, and interactions. The method can be adopted easily by the NMR community and integrated in research protocols, as CST takes only up to 2 h of computational time and the whole process can be carried out in the web browser using our cloud computing platform NMRtist (https://nmrtist.org). Although this is a technical paper presenting a solution to a common problem in biomolecular NMR spectroscopy, where approaches analogous to molecular replacement in X-ray crystallography are not in common use, it may have a more general impact on biochemical research by simplifying the use of NMR in situations where it was so far considered a (too) laborious method. Additionally, we believe that the results presented here may serve as guidance for NMR practitioners who use the NMRtist platform (Klukowski et al., 2023), helping them to assess the practical benefits of the recently proposed ARTINA method in a new context.

## Data Availability

Publicly available datasets were analyzed in this study. This data can be found here: ETH Research Collection: https://doi.org/10.3929/ethz-b-000568621.
