# Supplementary material for: Chemical shift transfer: an effective strategy for protein NMR assignment with ARTINA
Source: Front Mol Biosci. 2023 Oct 3;10:1244029. doi: 10.3389/fmolb.2023.1244029 (PMC10581199; doi:10.3389/fmolb.2023.1244029)
Supplement: Supplementary file 1 [file Table1.PDF]

**Supplementary Table S1.** Parameters used for protein alignment. Alignment of protein sequences for chemical shift transfer and scoring was performed using the Biopython library within Python 3.7.0. To yield an alignment score independent of sequence length and within the range [0, 1], each score was normalized by that of the alignment of the respective target protein to itself.

| | |
|---|---|
| **Aligner object** | Bio.Align.PairwiseAligner |
| **Alignment type** | Local |
| **Gap-opening score** | -10 |
| **Gap extension score** | -0.5 |
| **Substitution matrix** | BLOSUM45 |
