# Supplementary material for: Chemical shift transfer: an effective strategy for protein NMR assignment with ARTINA
Source: Front Mol Biosci. 2023 Oct 3;10:1244029. doi: 10.3389/fmolb.2023.1244029 (PMC10581199; doi:10.3389/fmolb.2023.1244029)
Supplement: Supplementary file 2 [file Table2.pdf]

Supplementary Table S2. Subsets of the NMR benchmark database used for experiments. Proteins are identified by PDB code where available, or otherwise by common abbreviation.

**Use in this work**	**Spectra**	**Proteins**	**Total proteins**
Assignment with synthetic data for transfer	^{13}C-HSQC, ^{13}C-HSQC (aromatic), ^{15}N-HSQC, ^{13}C-NOESY, ^{13}C-NOESY (aromatic), ^{15}N-NOESY	1SE9, 2JVD, 2K1G, 2K50, 2KFP, 2KOB, 2MDR, 2KHD, 2LTM, 2KBN, 2LAK, 1YEZ, 2KL6, 2JRM, 2RN7	15
Transfer from RefDB/BMRB	^{13}C-HSQC, ^{13}C-HSQC (aromatic), ^{15}N-HSQC, ^{13}C-NOESY, ^{13}C-NOESY (aromatic), ^{15}N-NOESY	1SE9, 2JVD, 2K1G, 2K50, 2KFP, 2KJR, 2KKL, 2KL5, 2KOB, 2LEA, 2LFI, 2LML, 2LND, 2LRH, 2LTL, 2LX7, 2MDR, 2HEQ, 2JN8, 2JT1, 2K3A, 2K52, 2K5V, 2KD0, 2KHD, 2KIF, 2KKZ, 2L06, 2L3B, 2L8V, 2LK2, 2LN3, 2LTM, 2LXU, 2M7U, 2MB0, ENTH, 2JQN, 2K3D, 2K5D, 2KBN, 2KD1, 2KK8, 2KPN, 2KRT, 2KVO, 2L1P, 2L3G, 2LAK, 2LF2, 2LL8, 2LNA, 2MA6, RHO, 1PQX, 1T0Y, 1YEZ, 2B3W, 2JXP, 2K0M, 2K57, 2K75, 2KCD, 2KCT, 2KL6, 2KZV, 2L33, 2LGH, 2M47, 2MK2, SH2, 2ERR, 2JRM, 2JVO, 2K1S, 2K53, 2KIW, 2M4F, 2MQL, 2N4B, 2RN7, 6FIP, 6GT7, 6SOW, KRAS4B, MH04	86
