# Supplementary material for: Chemical shift transfer: an effective strategy for protein NMR assignment with ARTINA
Source: Front Mol Biosci. 2023 Oct 3;10:1244029. doi: 10.3389/fmolb.2023.1244029 (PMC10581199; doi:10.3389/fmolb.2023.1244029)
Supplement: Supplementary file 3 [file Image2.pdf]

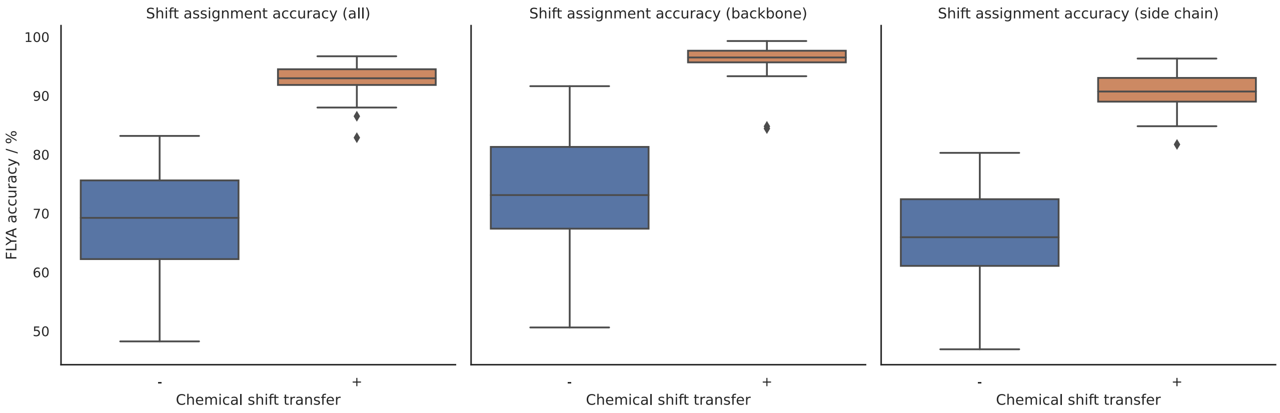

Supplementary Figure S2. Aggregated results of reference experiments using the subset of 15 proteins selected for the minimal set of spectra. "–" represents assignment without chemical shift transfer and "+" represents the ideal case of chemical shift transfer, using each protein's own assigned shift list as a source.
