# Supplementary material for: Chemical shift transfer: an effective strategy for protein NMR assignment with ARTINA
Source: Front Mol Biosci. 2023 Oct 3;10:1244029. doi: 10.3389/fmolb.2023.1244029 (PMC10581199; doi:10.3389/fmolb.2023.1244029)
Supplement: Supplementary file 4 [file Table3.PDF]

Supplementary Table S3. Source-target pairs selected from BMRB. Source proteins identified by BMRB entry ID, target proteins identified by PDB code where available, or otherwise by common abbreviation.

**Source**	**Target**
bmr7225	2JVD
bmr17706	2LEA
bmr17707	2LEA
bmr16805	2LL8
bmr18263	2LL8
bmr18561	2LND
bmr17613	2LRH
bmr18337	2LRH
bmr26801	2LTM
bmr25041	2MQL
bmr34290	6GT7
bmr19502	2MDR
