# Supplementary material for: Chemical shift transfer: an effective strategy for protein NMR assignment with ARTINA
Source: Front Mol Biosci. 2023 Oct 3;10:1244029. doi: 10.3389/fmolb.2023.1244029 (PMC10581199; doi:10.3389/fmolb.2023.1244029)
Supplement: Supplementary file 5 [file Image1.pdf]

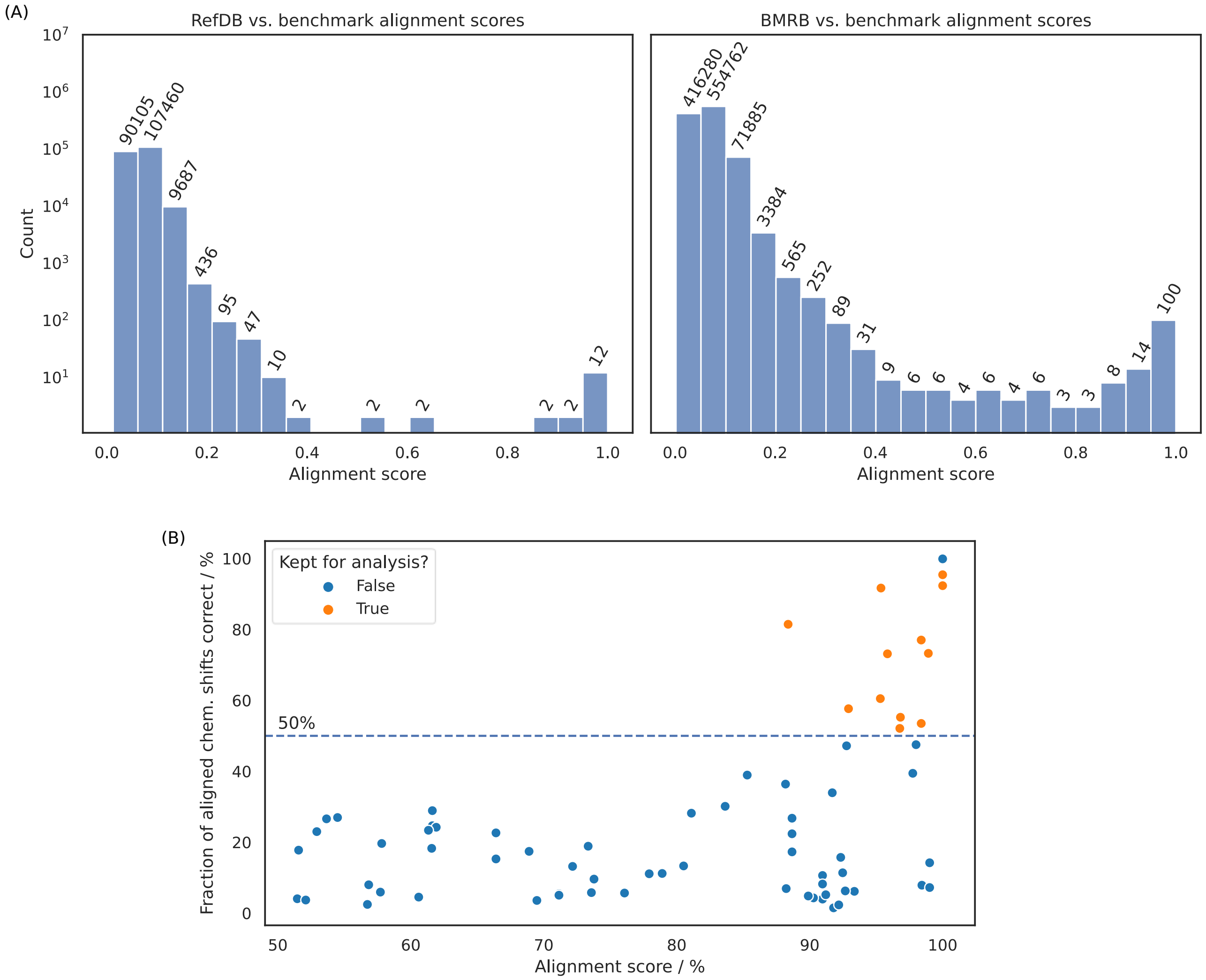

Supplementary Figure S1. Selection of data for homology-based experiments. **(A)** Histogram of all alignments between the benchmark database and RefDB (left) or BMRB (right). The number of pairs per bin is displayed above the corresponding bins. **(B)** All benchmark-BMRB alignments in detail, with the "ideal pairs" used for further analysis in orange. "Fraction of aligned chem. shifts correct" refers to the fraction of source shifts within a small tolerance (^{1}H: 0.03 ppm, ^{13}C/^{15}N: 0.4 ppm) of their target values after protein sequence and chemical shift alignment, regarding only the shifts appearing in both source and target. Only pairs with an alignment score above 80 % and a fraction of correct chemical shifts above 50 % were used, and all pairs at 100 % were discarded, leaving a total of 12 samples.
